# A safe teaching protocol of LRP (Laparoscopic Radical Prostatectomy)

**DOI:** 10.1590/S1677-5538.IBJU.2017.0137

**Published:** 2018

**Authors:** Marcos Tobias-Machado, Cristiano Linck Pazeto, Oseas Castro Neves-Neto, Igor Nunes-Silva, Hamilton de Campos Zampolli

**Affiliations:** 1 Departamento de Urologia, Faculdade de Medicina ABC, Santo André, SP, Brasil; 2 Instituto do Câncer Arnaldo Vieira de Carvalho – IAVC, São Paulo, SP, Brasil

**Keywords:** Prostatectomy, Education, Surgical Procedures, Operative

## Abstract

**Purpose:**

The LRP has a steep learning curve to obtain proficiency during which patient safety may be compromised. We present an adapted modular training system which purpose to optimize the learning curve and perform a safe surgery.

**Materials and Methods:**

A retrospective analysis of the LRP safe learning protocol applied during a fellowship program over eight years (2008-2015).

The surgery was divided in 12 steps and 5 levels of difficulty. A maximum time interval was stipulated in 240 minutes. After an adaptation, the fellows had 120 minutes to perform all the corresponding modules to its accumulated skill. The participants gradually and safely pass through the steps and difficulty levels. Surgeries performed by fellows were analyzed as a single group and compared to a prior series performed by tutor.

**Results:**

In eight years, 250 LRP were performed (25 per apprentice) during fellowship program and 150 procedures after completion. The baseline characteristics were comparable. Most cases operated were of intermediate risk. Mean operative time was longer in the fellow group when compared to the tutor (150 min). Mean estimated blood loss were similar among the groups. Functional and oncological outcomes were better in the Tutor's group. No conversion to open surgery was performed.

**Conclusions:**

The LRP safe learning protocol proved to be an effective method to optimize the learning curve and perform safe surgery. However, the tutor's functional and oncological results were better, showing that this is a procedure with a steep learning curve and proficiency demands more than 25 cases.

## INTRODUCTION

Robot-assisted laparoscopic prostatectomy (RALP) is the standard procedure for minimally invasive prostate cancer treatment in several countries including US (1-3). This approach emerged as an evolution of laparoscopic radical prostatectomy (LRP). However, the high cost involved with this technology remains a huge obstacle to its expansion in developing countries. As described previously, there are few units of *da Vinci Surgical System* ^®^ (Intuitive Surgical, EUA) in Brazil. So, the number of people covered by this technology is very limited. Additionally, most of Brazilian urologists have not had access to RALP to become proficient in this method.

In this context, with an increasing and inexorable trend to perform minimally invasive techniques, the LRP appears as an alternative that seems important to keep teaching. The LRP provides improved visualization of the prostatic anatomy, accurate dissection, decrease in length of hospital stay and lower blood loss, comparing to open approach. However, it is one of the most technically demanding procedures in Urology (4). Some aspects inherent of laparoscopy such as two-dimensional images, decreased tactile sensation and limited range of motion can explain its difficulty. Besides, the abilities required to LRP differs from those of open surgery and attempts to transfer skills are not effective. Thereby, there is a long learning curve that has limited its expansion.

some procedures may last too long, (especially when not assisted by experienced urologists) thus subjecting the patient to serious risks associated with positioning and pneumoperitoneum (5). For example Schuessler and colleagues first series reported lengthy operative duration (8 to 11 hours) and too long hospital stay (average 7.3 days) (6).

Thus, to optimize the learning curve and perform safe surgery, we present an adapted modular training system for fellows. The purpose is allowing a safe and optimal learning process dividing the steps of surgery by level of difficulty. The results and the effects of this model in the learning curve will be accessed and compared to the tutor performance.

## MATERIALS AND METHODS

This is a retrospective analysis of a LRP safe learning protocol. The 5-6 port LRP were performed during a 1-yr fellowship program in a Brazilian referral hospital. There were 10 fellows over a period of eight years (2008-2015).

The technique performed in the modular training was anterograde endoscopic extraperitoneal radical prostatectomy (Table-1). Based on previous study of Stolzenburg et al. (7), we divide the surgery in 12 steps on which were assigned levels of difficulty from 1 to 5, being 5 the most difficult.

**Table 1:** Steps of modular training.

No of step	Description of step	Module level
1	Umbilical incision, creation of extraperitoneal space and trocar placement	I
2	Pelvic lymphadenectomy	II
3	Exeresis of pre-prostatic fat, bilateral opening of endopelvic fascia and section of pubo-prostatic ligaments	I
4	Vascular control of dorsal vein complex (X-stitch) and control of retrograde blood flow (Hem-o-lock^®^)	III
5	Apical dissection	IV
6	Blunt retroprostatic dissection and incision of the posterior Denonvillie's fascia	III
7	Section of the bladder neck with anterior and lateral dissection	II
8	Dorsal bladder neck dissection	III
9	Identification and division of vasa deferentia and dissection of the seminal vesicles	III
10	Control of the prostatic pedicles/Nerve sparing procedure	III/V
11	Urethrovesical anastomosis (continuous 3-0 suture begins at 6 o'clock position and rises toward 12 o'clock bilaterally)	IV
12	Drainage with penrose drain, removal of the specimen and closure portals	I

All study participants were graduated urologists with prior experience in laparoscopic procedures (e.g.: nephrectomy, pyeloplasty) that did not include, however, pelvic procedures. When entering the program, all participants received training in dry-lab, including laparoscopic suturing techniques. The first 5 procedures were performed by the tutor with assistance from two fellows (one as holder camera and the other as first assistant). After this initial period, the tutor began to assist the trainees.

The tutor participated in all the procedures performed. A maximum time interval for surgery was stipulate in 240 minutes. Within this time, the trainees had 120 minutes to perform all the corresponding modules to its accumulated skill and at least, try those there are more complex. This initially settled time could have been subjectively extended if surgery was advancing properly. However, in case of no progression of the procedure, the tutor returns to assume. During the following operations, the fellows gradually and safely passed through steps. In the end of the training, participants could try to perform the entire procedure within the recommended time. There was no case selection. The neurovascular bundles preservation was considered the most difficult step (level V).

The cases operated by the fellows were analyzed as a single group and compared to a prior series of 200 cases performed by the tutor (8). The number of procedures per fellow was the same to reduce the skill bias and homogenize the sample. Additionally, the results obtained in the post fellow set were included in this analysis as a third group.

The mean operating time, transfusion rate, intraoperative complications, length of hospital stay, and positive surgical margins rates were analyzed. Clinical evaluation and PSA dosages were done at 45 days after surgery and every three months over first two years to assess functional and oncological outcomes.

Urinary continence was defined as 0-1 pads/day at 12 months of follow-up. Potency status was evaluated through the question 5 of the IIEF (ability to maintain penile rigidity and erection sufficient for intercourse). All detected postoperative complications were graded in Clavien-Dindo Classification (9). Continues variables were compared by paired t-test, while categorical variables were analyzed by chi-square test. The confidence interval was 95 % (p<0.05) for statistical significance. Informed consents were obtained by all patients and the study has been authorized by appropriate ethics committee (CAAE: 61327016.4.0000.0082).

## RESULTS

In eight years of study (2008-2015), the first 25 LRP of each fellow were included (totaling 250 procedures during the program). Also, the first 150 procedures after fellow were accounted (as independent practice). There was no difference between mean age, BMI and pelvic previous surgeries among the three groups (Table 2). Mean prostate volume in tutor and fellow group was 55g (18-200) and 46g (20-101), respectively (p=0.06). Most of patients were classified as intermediate-risk group (60% vs. 50%, respectively for tutor and fellows). A higher percentage of high-risk cases was found in the tutor group (p <0.001).

**Table 2:** Demographic data of our patients that underwent LRP comparing fellows during and after fellowship and expert results.

Criteria	Fellows (10)		Tutor	P value
	Fellow	After Fellow	Last cases	
No. of cases operated	250	150	200	
Age range (mean)	48-71 (65)	49-71 (64)	46-75 (65)	1.0
Prostate volume (mean)	20-101 (46)	20-89 (40)	18-200 (55)	0.06
D'Amico criteria (n/%)				
Low risk	75/30%	60/40%	20/10%	
Intermediate risk	125/50%	75/50%	120/60%	<0.001
High risk	50/20%	15/10%	60/30%	
BMI	28 (25-34)	29 (26-36)	30 (24-41)	0.5
Pelvic previous surgery	20%	22%	25%	0.09

Mean operative time (OT) was longer in the fellow group (241 min) and post- fellowship (249 min) compared to the tutor (150 min; p=0.03). Mean estimated blood loss was 350 ml (range 150-800) for the fellows, 300ml (range 200-600) for postfellowship and 250ml (range 100-500) for the tutor. Transfusion rates were similar between groups (2.8% vs 2% vs 1%; p=0.39). There were no grades IV and V complications and most were classified as grade I (Clavien-Dindo). Mean hospital length of stay was 2 days both for the fellow and for post- fellowship groups (range 1-7 and 1-3, respectively) and 1 day (range 1-3) for the tutor. Mean catheterization time was 12 days in the fellow group, 10 days in post-fellowship and 8 days in the tutor series (Table 3).

**Table 3:** Perioperative data comparing fellows during and after the fellowship period and tutor results.

Criteria			Fellows (10)		Tutor	P value
			Fellow	After Fellow	Last cases	
Operative time Range (mean)			181-302 (241)	199-300 (249)	134-239 (150)	0.03
Estimated blood loss Range (mean)			150-800 (350)	200-600 (300)	100-500 (250)	0.06
Transfusion rate (%)			2.8%	2%	1%	0.39
	**Complications (%)**					
		**Clavien-Dindo**				
		Grade I	14%	20%	20%	
		Grade II	4%	2%	2%	
		Grade III	2%	0	2%	0.8
		Grade IV	0	0	0	
		Grade V	0	0	0	
**Total**			**30%**	**22%**	**24%**	
Hospital stay (days)			1-7 (2)	1-3 (2)	1-3 (1)	0.3
Catheterization days Range (mean)			7-21 (12)	7-14 (10)	7-14 (8)	0.08

There were higher rates of continence (92% versus 98%, p=0.03) and potency (65% versus 80%, p=0.04) in the Tutor's group. The positive margin rate for pT2 was 20%, 15% and 10% and for pT3 was 35%, 30% and 25% among those groups, respectively (p=0.04). No conversion to open surgery was required (Table 4).

**Table 4:** Oncological and Functional outcomes.

Criteria		Fellows (10)		Tutor	P value
		Fellow	After Fellow	Last cases	
**Continence**					
	Yes	92%	92%	98%	0.03
	No	8%	8%	2%	
**Preservation of Potency**					
	Yes	65%	68%	80%	0.04
	No	35%	32%	20%	
**Positive Margins**					
	pT2	20%	15%	10%	0.04
	pT3	35%	30%	25%	

## DISCUSSION

The main objective of LRP safe learning protocol was fully achieved: there was a safe transfer of LRP skills during the fellowship period. The rate of perioperative complications was similar between the groups. There were no serious complications, deaths or conversions in all stages of the learning curve which reinforces the importance of this method.

The OT of the fellows group was higher, but the average time was less than 4 hours (the longest lasted about 5 hours). This relatively short time during the learning period reduces significantly the risks of metabolic complications associated with the surgical procedure and keeps patient safe.

There was a larger number of high risk patients in tutor group; however, when these high-risk cases were operated by fellows, the surgery proceeded without any significant complications. Prostate weight was comparable between groups and this is an important detail, since large glands decrease the visualization of the surgical field when performing the laparoscopic approach (10).

We detected a trend of higher transfusion rates in fellow group, although the estimated blood loss was similar. This occurred because the major bleedings, though few, occurred in this group. The explanation is the greater difficulty in controlling the prostate venous plexus and longer operative times. Still, these outcomes are acceptable and similar to other studies with a mentored fellowship program (7, 11-14).

Studies demonstrating decreased learning curve from a mentored learning model of LRP have been published since 2003 (11). In 2006, Stolzemburg and colleagues created the Leipzig method for learners without any experience and reached satisfactory results (9). In 2013, these authors presented the five year's follow-up with good oncologic outcomes (12). Therefore, modulated learning of LRP has proven to be effective in achieving reasonable early and midterm outcomes (7, 15). However, there are several reports of failure of other teaching methods (courses and shorter programs) to incorporate LRP in urologists practice (16).

One of the biggest challenges of this surgery is achieving negative surgical margins status with functional preservation. There is not only a learning curve but multiple curves for different variables. Although the primary goal was not the evaluation of our functional and oncological outcomes, the expert's results were better. These because the LC for these criteria was not met by the learners. According to the literature, LC for oncological outcomes reaches plateau at approximately 200 to 250 cases (17, 18) and the number of cases required to achieve proficiency ranges from 200 to 700 (19). The 25 cases operated by apprentice did not allow them to reach proficiency in these aspects. Nevertheless, this protocol presents a safe and effective learning method.

Regarding surgical steps, a previous study compared systematically the single steps of LRP with those of open approach. Although the anastomosis time decreased over time, it was considered the hardest and most demanding surgical step and its difficulty score remained high and stable (20). In our study, it was remarkable the difference in achieving proficiency in steps as Retzius access space, division of dorsal vein complex and dissection of seminal vesicles when compared to most difficult ones: preservation of neurovascular bundles and urethrovesical anastomosis. The choice of a bilateral continuous 3-0 monocryl suture to perform the vesicourethral anastomosis (Van Velthoven) improved results.

Finally, Another benefit of a LRP teaching model in a resident program was previous highlighted: even technically more demanding, there is a limited risk of mortality due to bleeding as in a kidney ablative surgery. Because of that, it could be a better model than nephrectomy to advance in laparoscopy training.

As a future perspective, previous published data demonstrated that experienced surgeons in laparoscopy can maintain their oncological and functional results even in the LC of RALP (21, 22). Although the RALP has overcame the LRP functional outcomes, we believe that in developing countries, such as Brazil, there's a relevant role to LRP, yet. Also, the transition to RALP will be helped by minimally invasive skills acquired.

The limitations of this study were its retrospective nature, small number of patients, the absence of mid and late oncological outcomes and temporal distance between tutor and fellow's series. However, it has achieved the central purpose assessing the feasibility and safety of this learning method.

## CONCLUSIONS

The LRP safe learning protocol proved to be an effective method to optimize the learning curve and perform safe surgery. However, the tutor's functional and oncological results were better, showing that this is a procedure with a steep learning curve and proficiency demands more than 25 cases.

## Abbreviations

LC: Learning Curve
LRP: Laparoscopic radical prostatectomy
RALP: Robot-assisted laparoscopic prostatectomy
OT: Operative time
IIEF: International Index of Erectile Function
